# Cholinergic Neuromodulation Changes Phase Response Curve Shape and Type in Cortical Pyramidal Neurons

**DOI:** 10.1371/journal.pone.0003947

**Published:** 2008-12-16

**Authors:** Klaus M. Stiefel, Boris S. Gutkin, Terrence J. Sejnowski

**Affiliations:** 1 Howard Hughes Medical Institute, The Salk Institute for Biological Studies, La Jolla, California, United States of America; 2 Group for Neural Theory, DEC, ENS, College-de-France, CNRS, Paris, France; 3 Division of Biological Sciences, University of California San Diego, La Jolla, California, United States of America; University of Pittsburgh, United States of America

## Abstract

Spike generation in cortical neurons depends on the interplay between diverse intrinsic conductances. The phase response curve (PRC) is a measure of the spike time shift caused by perturbations of the membrane potential as a function of the phase of the spike cycle of a neuron. Near the rheobase, purely positive (type I) phase-response curves are associated with an onset of repetitive firing through a saddle-node bifurcation, whereas biphasic (type II) phase-response curves point towards a transition based on a Hopf-Andronov bifurcation. In recordings from layer 2/3 pyramidal neurons in cortical slices, cholinergic action, consistent with down-regulation of slow voltage-dependent potassium currents such as the M-current, switched the PRC from type II to type I. This is the first report showing that cholinergic neuromodulation may cause a qualitative switch in the PRCs type implying a change in the fundamental dynamical mechanism of spike generation.

## Introduction

The ability of neurons to synchronize their spiking activity depends on their mutual synaptic connectivity and their intrinsic properties. The intrinsic properties can be revealed by the phase response curve (PRC) [1], defined as the spike time shift caused by a small perturbation of the membrane potential as a function of the time of the perturbation during the spike cycle. The PRC allows a classification of neurons into two fundamentally different classes with regard to their spiking behavior [1].

In neurons with a purely positive, or *type I*, PRC, perturbations at every phase of the oscillatory cycle cause a time-advance of the next spike. By convention for a PRC a time-advance is plotted in the positive and time-delay in the negative directions. For neuronal models with such purely positive PRCs measured at relatively low firing rates, the transition from rest to tonic spiking comes about generically through a *saddle-node bifurcation* [1]. The resulting dynamics imply a general absence of subthreshold oscillations and the onset frequency of firing is arbitrarily low. The firing frequency – injected current relationship is roughly linear and the action potentials are all-or-none and well separated from subthreshold responses. No bistability near the rheobase and no hysteresis is apparent for this type of membrane excitability. A large number of biophysical models of cortical pyramidal neurons has type I excitability [1], [2], [3].

In neurons with a biphasic, or *type II*, PRC, perturbations at early phase positions cause a delay of the next spike, whereas late perturbations cause an acceleration of the spike time. These neurons tend to show subthreshold oscillations and a finite onset frequency of firing. The transition from rest to tonic spiking occurs via a *subcritical Hopf bifurcation*. The firing frequency vs. injected current relationship is non-linear (has a discontinuity near the onset of firing) and the action potential amplitude can be graded. For certain parameter regimes bistability between a quiescent and a tonically firing behavior can appear. The Hodgkin-Huxley equations with the original parameters describing spiking in the squid giant axon [4] are type II.

The PRC type is interesting to ascertain since it can provide insights into the neuron's synchronization behavior. Theory suggested that excitatory coupling tends to desynchronize networks of neurons with type I PRCS [1], and synchronize neurons that have significant negative regions in the PRC (Type II), and/or with PRC that have a strong right skew (see Gutkin et al. [5] for further discussion). Hence the PRC links the biophysics of spike generation in neurons and their behavior in large networks.

There have been a number of experimental reports of PRCs measured in cortical neurons. Reyes and Fetz [6] measured PRCs in layer V cortical pyramidal neurons and found that they had a characteristically skewed shape and appeared to be mostly type I (no negative portions in the PRC). Netoff *et al.* [7] identified PRCs in stellate cells in the enthorhinal cortex using the dynamic clamp technique; their results show PRCs qualitatively similar to those found by Reyes and Fetz [6] but with a small negative component at the beginning of the firing cycle. Galan *et al.* [8] measured PRCs from mitral cells in the olfactory bulb and found PRCs with clear negative portion at the beginning of the cells firing cycle. Thus, motor-cortical pyramidal neurons appear to be of type I, entorhinal neurons are “weakly” type II and olfactory bulb neurons are solidly type II. Further recent data shows that certain CA3 pyramidal neurons appear to be type II [9]. In vitro recordings in the somatosensory cortex indicate that fast spiking interneurons are of type II [10]. A recent in vitro study showed that pyramidal neurons in layer V are predominantly type I, while layer 2/3 pyramids are predominantly type II [11] depending on the basal firing rate of the neuron as predicted in Gutkin et al [5]. These earlier studies, with the notable exception of the last, have not asked the question of what intrinsic cellular and functional mechanisms might control the change between the two PRC classes in a given cell or in mathematical terms what might cause a transition from one bifurcation class to another.

Here we address this issue and focus on the cholinergic modulation of slow potassium currents. Acetylcholine is a central nervous system neuromodulator that is of significant behavioral and functional importance. The level of acetylcholine is elevated during awake, vigilant states and it is associated with a globally desynchronized EEG, increased power in higher frequency bands [12] and increased synaptic plasticity [13], [14]. Most *in vitro* models of fast cortical oscillations use cholinergic neuromodulation [15], [16]. Whereas data shows that acetylcholine down-regulates slow potassium currents that underlie spike frequency adaptation and after-hyperpolarization [17], previous theoretical work showed that modulation of such currents can convert neurons from type I to type II [2]. The switch should primarily depend on K-currents that activate below the firing threshold of the cell, such as the muscarine-sensitive M-current (associated with the CHRM gene). This points to a link between the dynamics of spike generation at the cell level and cholinergic neuromodulation on the systemic level. Hence we experimentally explored the possible connection between cholinergically down-regulated potassium currents and PRC type of cortical pyramidal neurons.

## Methods

### Electrophysiology

We recorded from layer II/III pyramidal neurons in the slices of the mouse visual cortex. All animal experiments were done in accordance with ethical guidelines of the Salk Institute. Mice (B6D21/Hsd B6, “black 6”, Harlan, San Diego, age P28 to P35) were anesthetized with halothane and decapitated. The occipital forebrain was removed and glued to a plastic block. Coronal slices of the visual cortex (300 µm) were cut with a Series 1000 Vibratome (Pelco) or a custom-made slicer in ice-cold artificial cerebrospinal fluid (ACSF , NaCl 125 mM, KCl 2.5 mM, NaH_2_PO_4_ 1.25 mM, NaHCO_3_ 25 mM, CaCl_2_ 2mM, MgCl_2_ 1.3 mM, Dextrose 10 mM). Slices were allowed to recover in ACSF at 35°C for at least 30 minutes before the start of recordings.

Recordings were performed under IR-DIC videomicroscopy [18] in oxygenated ACSF (flow 3 ml/min) at 32°C. Whole-cell patch clamp recordings were performed with electrodes ranging from 5 to 8 MΩ. The pipette solution contained KMeSO_4_ 140 mM, HEPES 10 mM, NaCl 1.5 mM and EGTA 0.1 mM.

The voltage signal was recorded with an Axoclamp-2A amplifier (Axon Instruments, Foster City, CA, USA), low pass filtered at 30 kHz and digitized at 32 kHz with a PCI-MIO-16E-4 DAQ board (National Instruments, Austin, TX, USA). Data acquisition software was custom written in Lab View 6.1 (National Instruments, Austin, TX, USA).

Glutamatergic ionotropic synaptic transmission was blocked with DNQX (20 µM) and APV (50 µM) in all experiments. GABAergic ionotropic transmission was blocked with biccuculine (10 µM) in the majority of experiments. All drugs were purchased from Sigma (Dallas, TX, USA).

Action potential firing was evoked by injecting DC current via the patch-electrode, from −10 pA in the case of spontaneous spiking caused by the application of carbachol, to 150 pA. Continuous stretches spanning 32 seconds were recorded. In addition, short (10 to 20 ms) depolarizing (10 to 30 pA) current pulses were injected every 100 to (in most cases) 1000 ms to perturb the regular spiking process. Experiments were continued after at least 5 minutes after bath application of the cholinergic agonist carbachol (10, 20 or 50 µM). DC current injection was adjusted to achieve similar firing rates as before drug application.

**Figure 1:**
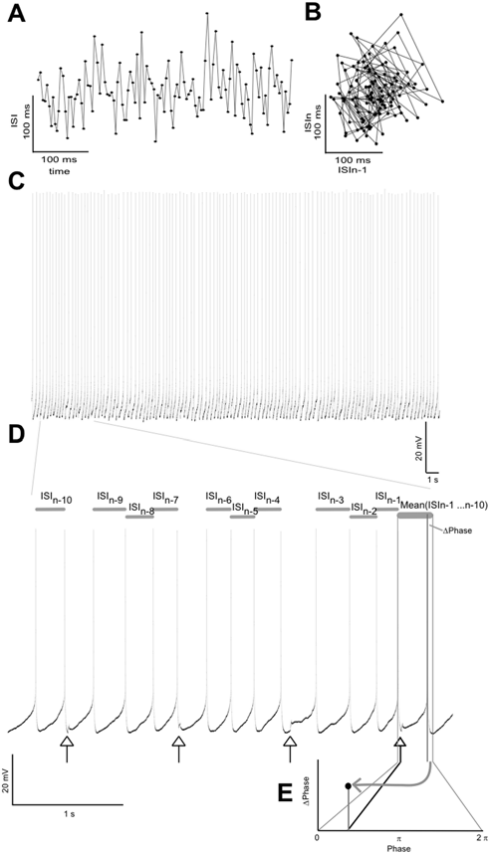
Determining PRCs from the perturbations of tonically spiking neurons. (A) ISIs during 500 ms of spiking recorded in a layer II pyramidal neuron. (B) ISI return map (ISI_n_ vs. ISI_n+1_). The lack of a geometrical structure points to the absence of a higher-order periodicity giving rise to the ISI fluctuations. (C) Voltage trace during 32 s of continuous spiking. (D) Expansion of (A). The perturbing pulses are indicated by arrows. The average duration of the 10 preceding ISIs (excluding other ISIs containing pulses) was subtracted from the ISI containing a perturbing pulse. The difference is Δphase. (E) The phase of the perturbing pulse (x-axis) and Δphase (y-axis) were plotted to determine the PRC.

Input resistance, resting potential and spike amplitude were monitored throughout the experiment and data acquired after significant changes in these parameters (independently of pharmacological manipulations) were discarded. Also, when entrainment of the spikes by the current pulses was observed, all the data from that neuron was discarded. We further monitored that there were no long-lasting trends in the cells firing rate (or equivalently inter-spike interval duration), see Figure 1A,B. Finally, we excluded cells which spiked in doublets (this occurred in 2 cells after the application of carbachol), since such a spiking pattern invalidates the analysis used here.

### Data Analysis

**Figure 2:**
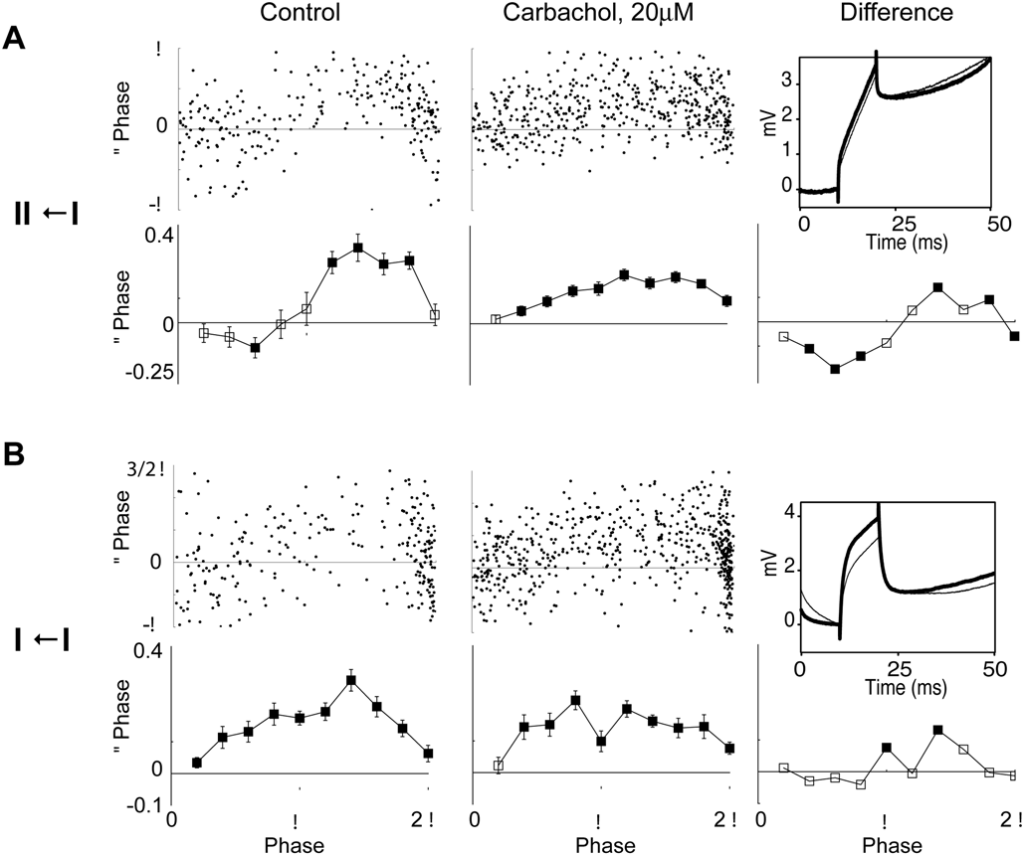
Change of PRCs due to cholinergic neuromodulation in single neurons. Left column: control conditions. Center column: bath application of carbachol. Right column: difference between the charbachol and control conditions. Top plots: raw data. Bottom plots: mean±s.e.m. of binned data. Filled symbols: significant changes in comparison to the ISIs without a perturbation. Data from example neurons (A) showing a transition from a type II to a type I PRC and (B) remaining with a type I PRC. Insets: voltage averages of the perturbing pulses in the absence (thin line) and presence (thick line) of carbachol. Average amplitudes were (A) 3.2/3.5 mV (54/60 sweeps) and (B) 3.2/3.8 mV (67/67 sweeps).

Spike times were determined by searching for upward threshold crossings (−20 mV) of the voltage trace. The analysis is illustrated in Figure 1C,D. From these spike times (*t*), the interspike intervals (ISIs) were determines as the difference between *t_n_* and *t_n+1_*. The change of the ISI length (Δt ISI) for ISIs containing a perturbing pulse was determined by subtracting its length from the mean of the lengths of the 10 previous ISIs. We determined the PRC by plotting *Δt* ISI/ISI of all ISIs containing a perturbing pulse as a function of the relative time of the pulse, with ISI duration normalized to 0 to 2 π (Fig. 2). We then binned the values for *Δt* ISI/ISI, according to the relative pulse time (10 bins/2 π), for single cells and across cells and calculated the mean and standard deviation for each bin. Spike time shifts were considered significant when a mean difference test, based on Student's t-test, gave p<0.01.

Cellular properties V_rest_, R_in_ and spike frequency adaptation (defined as as ISI_1_/ISI_3_ in a train of at least 4 spikes in response to the first 500 ms current pulse) were measured at the beginning of the experiment and after cholinergic effects had stabilized, before the determination of the PRCs under cholinergic conditions. The values under control and cholinergic conditions were consistent from trial to trial. As they were quite variable in between neurons, the differences across these populations were not significant (mean difference test as above).

## Results

We recorded from 9 cortical layer II/III pyramidal neurons and determined their PRCs. On average, 2020 ISI without and 570 ISIs with perturbing current pulses were used for the determination of the PRCs. Of these neurons, 5 initially had a type I (purely positive) and 4 a type II (biphasic) PRC.

Out of 4 type II neurons, all changed to type I after bath-application of the cholinergic agonist carbachol, see example in Figure 2A. In these 4 neurons, carbachol increased (toward positive) the PRC at early phases (bins 1/5 π to 3/5 π) and decreased at it late phases (>9/5 π).

Carbachol did not change the PRC type in any of the 5 type I neurons, Figure 2B. The increases (>9/5 π) and decreases (1/5 π to 2/5 π, 4/5 π to π, 6/5 π to 7/5 π) of the PRC as a result of carbachol application did not show a phase-dependent trend.

We wanted to ascertain if the changes observed in the PRCs were due to the cholinergic down-regulation of slow voltage-dependent potassium currents and not due to changes in the stimulus amplitude. During our experiments, current pulse amplitude was kept constant, which lead to a slightly higher voltage pulse amplitude when the neurons were under the influence of carbachol. The voltage amplitude increased slightly as one would expect when the cross-membrane conductance is decreased by action of carbachol (Fig. 2, inset). Our numerical investigations [19] have shown that moderate increases in the pulse amplitude, such as observed in the experiment, lead to a scaling of the PRC amplitude, but not a change in type of the PRC.

**Figure 3:**
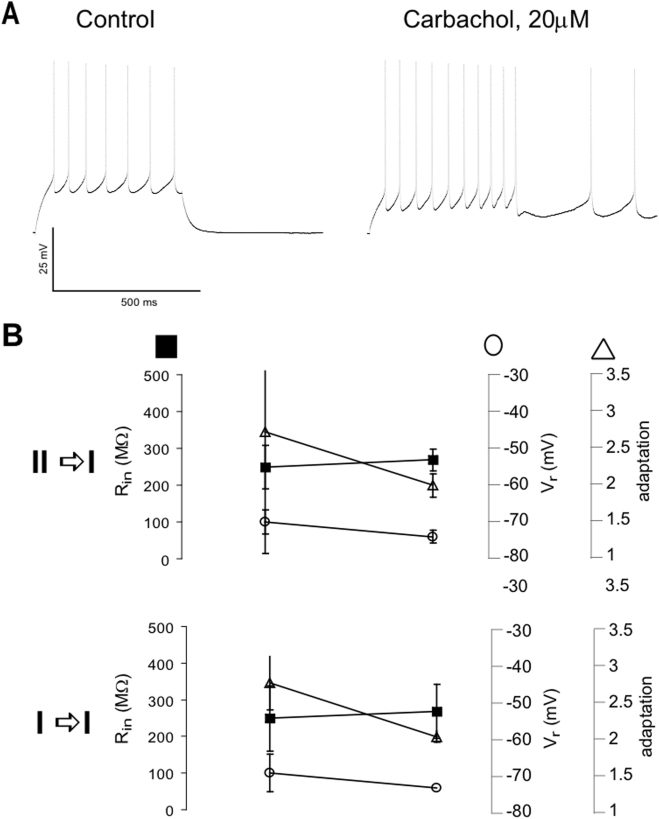
Electrophysiological properties and excitability in pyramidal neurons in the absence (left column) and presence (right column) of carbachol. (A) Spiking in response to the injection of a current pulse (500 ms). (B) Change of V_rest_, R_in_ and adaptation in response to the bath-application of carbachol in neurons which showed a transition from type II to type I PRC (top) and in neurons which did not (bottom).

We then asked if the changes in the PRC could be correlated with alteration in basic physiological properties of the neurons recorded. We saw that in 4 of the type II neurons, carbachol could induce persistent spiking that continued after a supra-threshold current injection (500 ms) (Fig. 3A, right). This would be consistent with carbachol down-regulating slow potassium currents that are responsible for controlling the neurons excitability. The application of carbachol also changed the input resistance (R_in_), resting potential (V_r_) and adaptation (see Methods). The results are summarized in Figure 3B. In the type II neurons that changed to type I in response to carbachol, R_in_ on average increased from 195 to 296 MΩ, V_r_ from −76.5 to −63.5 mV and the adaptation decreased from 3.05 to 1.04. In neurons with a type I PRC before and after the application of carbachol, R_in_ increased from 249 to 269 MΩ and V_r_ and the adaptation decreased from −69.9 to −74 mV and 1.72 to 0.99, respectively. When pooling all data, R_in_ increased from 229 to 318 MΩ, V_r_ from −71.8 to −67 mV and the adaptation decreased from 1.99 to 1.09. These results show that a specific combination of effects on R_in_, V_r_ and adaptation (increase, increase and decrease) is correlated with a switch from type II to type I PRC. However we could not tell if such a pattern is sufficient to explain or predict the changes in the PRC type. While from trial to trail the changes in the above parameters due to carbachol were consistent, we could not ascertain the significance of such changes across the cell population due to strong variability between neurons.

**Figure 4:**
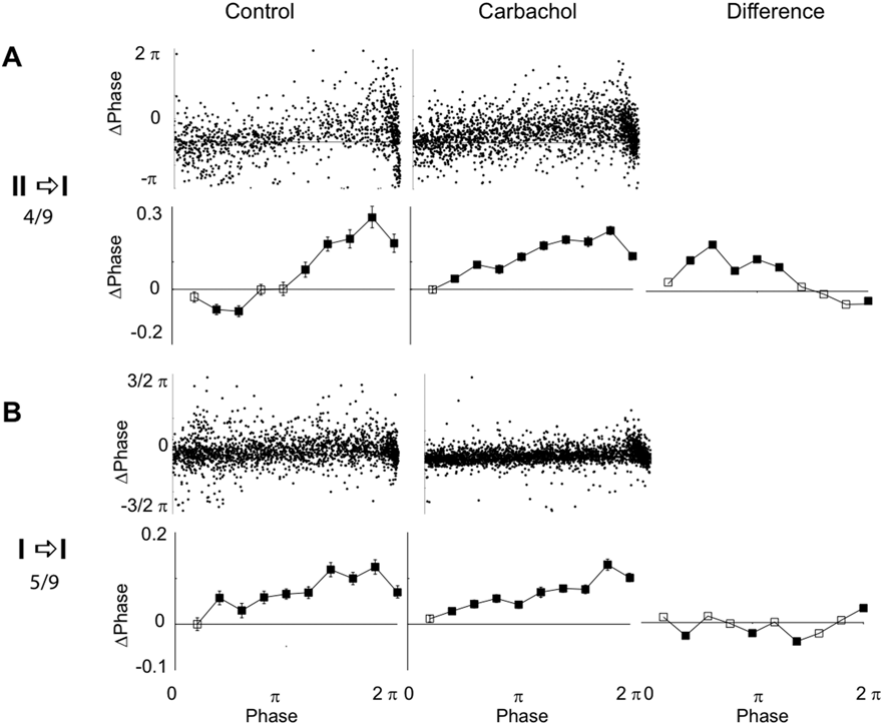
PRCs averaged over all neurons showing (A) a transition from a type II to a type I PRC in response to the bath-application of carbachol or (B) remaining with a type I PRC. Figure conventions as in Fig. 3.

We then wanted to ascertain that the PRC change due to carbachol could be seen across the whole cell population. When pooling the data from all neurons that showed the type II to I transition, we found that the PRC increased in early phases and decreased in the late phases, Figure 4A. In the averaged PRC for the I→I cells no trend in the PRC change was seen, yet several bins were significantly decreased by carbachol, Figure 4B. The latter was consistent with a decreased skew of the PRC.

**Figure 5:**
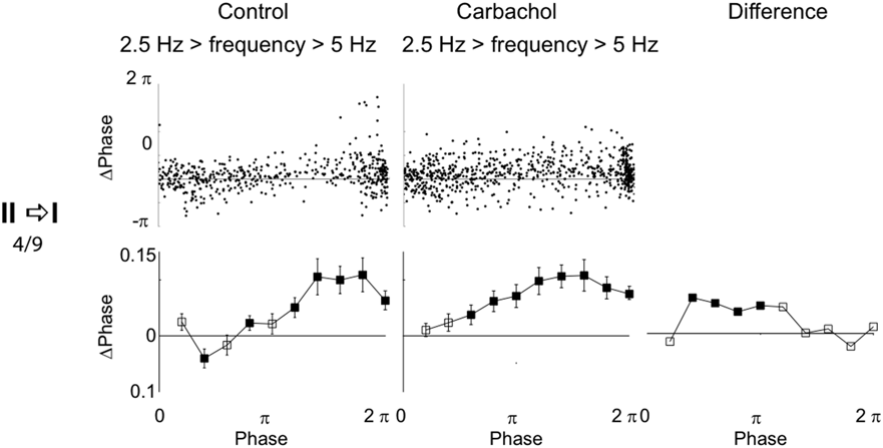
Charbachol induced PRC type I switch with mean firing rate normalized. PRCs averaged over all neurons showing a transition from a type II to a type I PRC in response to the bath-application of carbachol, but only including ISIs ranging from 400 (2.5 Hz) to 200 ms (5 Hz). Figure conventions as in Fig. 3.

When the firing frequency increases, the PRC of a neuron can shift from type II to type I (e.g. see [11]). This may occur when the time constants of the adaptation current responsible for type II behavior becomes too slow in relation to the spiking dynamics [5]. We thus had to assure that the observed switch in PRC type was not due to a higher firing frequency during the PRC determination under carbachol (2.6 vs. 4.9 Hz of the ISIs with a perturbation). We repeated the analysis including only ISIs ranging from 2.5 (400 ms) to 5 Hz (200 ms). The average firing frequencies of the ISIs included in this analysis was 3.8/4.1 Hz, and the qualitative switch of PRC type was still observed (Fig. 5).

## Discussion

We have shown that cortical pyramidal neurons can switch their PRC type, and thus most likely the type of bifurcation leading to spiking in response to cholinergic neuromodulation. This is the first demonstration of a qualitative switch in the basic principle underlying the transition from rest to action potential firing in any cell type. In the neurons that initially had a type II PRC it changed to type I after the application of carbachol. It did not change in the 5 neurons with initially a type I PRC. No case of a switch from type I to type II in response to carbachol was observed.

We observed heterogeneity in the properties PRC of cortical pyramidal neurons. Not all the recorded neurons changed their PRC type. A similar heterogeneity was also observed in the values of the neurophysiological parameters R_in_, V_r_ and adaptation and their changes induced by acetylcholine.

Furthermore, in some neurons the concentrations of carbachol used induced persistent firing that lasted after the current injection stimulus was removed. We suggest that such persistent firing is consistent with carbachol blocking currents that control the neurons excitability and possibly uncovering slow depolarizing currents. For example, effects we observed might be due the persistent sodium current that would activate slowly with spiking and persistently keep the cell above the firing threshold for an extended period after the current injection is removed. We note here that this kind of persistent firing appears to be dynamically different than the bistability that is associated with type II dynamics. For the latter no persistent depolarizing currents are required and one could switch between the quiescent and firing states by only a brief stimulus. Finally, numerical work shows that persistent sodium currents cannot explain type II behavior (results not shown).

The fact that type II neurons showing a strong carbachol-dependent decrease in spike-frequency adaptation switched their PRC type is consistent with the theoretical finding that decreasing an adaptation current can switch the type of bifurcation leading from rest to tonic spiking [2]. We suggest that the cholinergic decrease in a potassium current, possibly I_M_, was responsible for the transition from type II to type I. In fact, the cholinergic agonist we used acts through the muscarinic metabotropic acetylcholine receptors [20] that in turn control a number of cells parameters. Carbachol effects include the down-regulation of a number of slow K-currents as well as possibly non-specific currents such as the mixed ion current generating the leak. In principle all of those could be involved in the PRC switch we observed. However previous theoretical work showed that slow K-currents that depend on occurrence of spikes to activate (e.g. the I_KAHP(Ca)_) are not sufficient to cause the switch [2]. We also conducted a more careful numerical study of the possible carbachol effects on numerous currents as well as change is in the input resistance etc [19]. Our results also show that the M-current down-regulation is sufficient to cause the switch. At this point we cannot completely exclude that down-regulating other low-voltage activated potassium currents may not cause the observed effect, hence we are pursuing further studies with more selective M-current blockers. However the main results stands: cholinergic modulation consistent with down-regulation of the muscarine sensitive slow K-current converts type II neurons into type I.

Finally, in the type I neurons a decrease of adaptation was observed, but they were already of the PRC type associated with a small amount of adaptation current and their PRC type also did not change.

Given that about half of the pyramidal neurons in a cortical column switch their PRC type, would likely influence the synchronization properties of the neuronal network. Acetylcholine is known to promote the appearance of oscillations in the gamma-frequency range in vitro [21]. The switch in the spike generating dynamics, observed experimentally as a change in the PRC type, may be an important factor in this change.

An important question is what the above results mean for the synchronization of neurons in the cortex *in vivo*. This has to be addressed in the context of the complete cortical network, which in addition to the pyramidal neurons, contains a number of types of GABAergic interneurons. Different types of synchronous oscillations (delta, theta, gamma) result from different dynamics involving different currents and cell types [22], [23]. The gamma oscillations, which are evoked by elevated acetylcholine [21] are driven by synchronized interneural networks [15]. These interneurons then entrain the pyramids. Without interneural help, a network of reciprocally excitatory connected layer II pyramidal neurons would not synchronize when the acetylcholine concentration is high. This is predicted by theoretical results which show that neurons with a type I PRC (like layer II pyramids under the influence of acetylcholine) coupled with excitatory synapses don't synchronize [2]. On the other hand when such neurons are type II (e.g. without carbachol) they are predicted to synchronize and particularly well at lower firing rates.

The picture which thus emerges is that networks of cortical layer II pyramidal neurons synchronize well when subjected to no acetylcholine and even better when exposed to low concentrations of this neuromodulator [19]. This type of synchronization breaks down when the cholinergic neuromodulation becomes strong. Under such conditions, the pyramidal neurons are entrained into a gamma-rhythm by inhibitory interneurons.

This picture is consistent with the patchy and local nature of gamma oscillations, into which pyramidal neurons are forced by local interneural circuits, and the more global nature of low-frequency oscillations (delta, theta), which result from the properties of the pyramidal neurons and their networks themselves [5], [22], [23].

These results raise the possibility of a link between neuromodulation of the spike-generating process of individual neurons and changes in the large-scale network behavior and associated cognitive states in the mammalian cortex.
